# Deep learning based localisation and classification of gamma photon interactions in thick nanocomposite and ceramic monolithic scintillators

**DOI:** 10.1038/s41598-025-13339-y

**Published:** 2025-08-05

**Authors:** Mushen Shen, Ragy Abraham, Elise Cribbin, Harrison Gregor, Mitra Safavi-Naeini, Daniel Franklin

**Affiliations:** 1 School of Electrical and Data Engineering, University of Technology Sydney, Sydney, NSW Australia; 2 Australian Nuclear Science and Technology Organisation (ANSTO), Lucas Heights, NSW Australia

**Keywords:** Biomedical engineering, Photonic devices, Imaging techniques

## Abstract

Accurate localisation of the first point of interaction (FPoI) of incident gamma photons in monolithic scintillators is crucial for many radiation-based imaging applications - in particular, accurate estimation of the lines of response in positron emission tomography (PET). This is particularly challenging in thick nanocomposite and ceramic scintillator materials, which exhibit high levels of Rayleigh scattering compared to monocrystalline scintillators. In this work, we evaluate deep neural network-based approaches for (1) classifying the mode of photon interaction using an InceptionNet-based classifier and (2) accurately estimating the location of the FPoI based on scintillation photon distributions in several monolithic nanocomposite and ceramic scintillators using both CNN- and InceptionNet-based regression networks. The classifier was able to correctly categorise single-energy deposition events with an accuracy $\ge$ 90.1%, two-deposition interactions with an accuracy $\ge$ 77.6% and three-plus deposition interactions with an accuracy $\ge$ 66.7%. Across the evaluated materials, median total localisation error ranged from 0.58 mm to 2.91 mm with the CNN and 0.59 mm to 2.10 mm with InceptionNet, assuming 50% detector quantum efficiency. Localisation in nanocomposites using the InceptionNet-based regression network improved the most relative to previously-reported results based on classical techniques, in some cases approaching the accuracy achieved with ceramic scintillators.

## Introduction

Low-cost scintillator materials are attracting increasing interest for their potential use in radiation detection and imaging applications, including positron emission tomography (PET)^1,2^. Nanocomposite and transparent polycrystalline ceramic scintillators, despite having generally inferior optical and physical properties compared to monocrystalline scintillators, offer many advantages, including lower material costs and improved manufacturability^3,4^. This is particularly important for applications requiring large scintillator volumes, such as in monolithic PET detector modules, since the production of large, uniform monocrystals of high-performance scintillator materials such as cerium-doped lutetium-yttrium oxyorthosilicate (LYSO:Ce) is technically challenging and very expensive^5^.

Estimating the location of the first point of interaction (FPoI) between the emitted gamma photon and the scintillator is the initial step in utilising a monolithic scintillator in PET. Following energy windowing, coincident pairs of annihilation photons are identified and lines of response are formed between these locations and used to construct a sinogram for image reconstruction^6^. Some of the emitted photons are absorbed, lost or scattered within the patient; models for these processes (based on patient CT images) inform attenuation, activity normalisation and scatter correction algorithms. The energy acceptance window may also be broadened to include Compton-scattered photons, enabling the creation of hybrid PET-Compton imaging systems, which can offer improved signal to noise ratio compared to pure PET systems^7,8^.

The accuracy of this localisation is a critical factor in determining PET system performance. If one or more faces of the scintillator are covered in pixellated optical photodetector arrays, the distribution of detected optical photons can be used to estimate this location in three dimensions. However, this process is complicated by the large variety of possible types of energy-depositing interactions that can occur in the scintillator, ranging from a single photoelectric absorption of the 511 keV gamma photon to one or more Compton interactions, followed by either a final photoelectric absorption of the scattered photon or its escape from the scintillator. Each of these situations results in different optical photon distributions^9^.

Several approaches can be used for FPoI localisation. One method involves fitting a parametric analytic model of the optical photon distribution as a function of the initial point of interaction to the observed pattern and iteratively minimising the error between them to yield the coordinates of the point of interaction. This approach is challenging due to the many different modes of photon-matter interaction, as well as internal optical reflections, especially at the edge of the scintillator block^10^. Nevertheless, in previous work, we successfully utilised this approach to optimise the thickness of monolithic scintillators fabricated from nanocomposite and polycrystalline ceramics, maximising the probability of localising the point of interaction to within a specified threshold^11,12^. There has also been growing interest in the use of deep neural networks for localisation, leveraging their ability to learn complex nonlinear relationships between the system inputs and outputs given sufficient training data. Such training data can be obtained from Monte Carlo simulations, which is computationally feasible on modern computer hardware^13^.

Several previous studies have investigated the use of deep neural networks for FPoI localisation in the context of monocrystalline scintillator materials such as LYSO:Ce. Sanaat et al. evaluated the precision of a multilayer perceptron neural network to estimate the point of interaction in a monolithic LYSO:Ce scintillator coupled to a 12$\times$12 array of SiPM detectors, achieving localisation with a maximum error in depth of interaction (DoI) of 8.7% at interaction depths under 7 mm using a four-layer neural network^14^. Jalibarthi et al. employed a 10-layer deep-residual convolutional neural network to estimate event position in a 7.2 cm long annular monolithic PET scanner and showed that this approach outperforms the classical centre of mass (CoM) algorithm^15^. Carra et al. utilised several specialised neural networks to simultaneously estimate event positioning and timestamping, achieving an error of 0.78 mm FWHM on the (*x*, *y*) plane and 1.2 mm FWHM in the depth-of-interaction dimension, with 156 ps coincidence timing resolution on a 25 mm $\times$ 25 mm $\times$ 8 mm LYSO monolithic scintillator block^16^.

Despite the potential of deep neural networks for FPoI localisation, their performance in multi-centimeter thick nanocomposite and transparent ceramic materials remains unexplored. In nanocomposites, the difference in refractive indices of the nanoscintillator and the polymer matrix increases Rayleigh scattering - a phenomenon where optical photons are elastically scattered by particles or irregularities that are much smaller than its wavelength. Similarly, transparent polycrystalline ceramics suffer more from Rayleigh scattering than monocrystalline materials. This scattering results in a weaker and more diffuse optical photon pattern at the photodetector as the scintillator thickness increases. Although thicker scintillators improve sensitivity to incident gamma photons, they also degrade localisation accuracy due to the cumulative effects of Rayleigh scattering. Nonetheless, it is reasonable to expect that a deep neural network can account for these scattering effects on the optical photon distribution, providing an accurate estimate of the FPoI even in thick monolithic scintillator blocks.

In this work, we demonstrate the feasibility of using deep neural networks to localise the FPoI of gamma ray interactions in five thick nanocomposite scintillators (LaBr3:Ce-polystyrene, Gd2O3-polyvinyl toluene, LaF3:Ce-polystyrene, LaF3:Ce-oleic acid, and YAG:Ce-polystyrene) and four thick ceramic scintillators (GAGG:Ce, GLuGAG:Ce, GYGAG:Ce, and LuAG:Pr). Additionally, we demonstrate the classification of detected events by the type of interaction which has occurred.

## Materials and methods

This section describes the methods used to estimate the type and location of the first point of interaction of 511 keV gamma photons in monolithic scintillator blocks. The methodology involves three main steps:

- Generation of training data through Monte Carlo simulations;
- Development and training of neural networks for interaction classification and FPoI localisation; and
- Evaluation of network performance.

First, a large body of training data is generated by simulating the emission of 511 keV gamma photons with random position and orientation towards each of the nine evaluated scintillator materials using the Geant4 Application for Tomographic Emission (GATE) Monte Carlo simulation platform^17,18^. For each 511 keV gamma photon interacting with the slab under test, the first point of interaction between the incident gamma photon and the scintillator slab, as well as the resulting optical photon distribution on the optical photodetector plane and the mode/modes of interaction are recorded. The simulated photon distributions are then used to train three deep neural networks - one for interaction type classification, and two alternative regression networks for event localisation.

**Figure 1:**
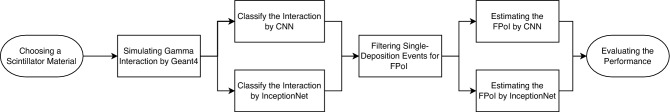
Flow chart illustrating the proposed approach.

A more detailed illustration is represented in Fig. 1.

In this work, the InceptionNet model is used to classify the type of interaction, while two regression networks - one based on a convolutional neural network (CNN) and the other based on a modified InceptionNet - are utilised for the localisation of the FPoI. InceptionNet is an advanced convolutional neural network (CNN) architecture originally developed by Google for computer vision problems^19^. Unlike classical CNNs that use fixed-size convolutional filters, InceptionNet introduces “inception modules” that perform parallel convolutions with filters of different sizes (e.g., 1$\times$1, 3$\times$3, and 5$\times$5) where their outputs are concatenated along the channel dimension. In this approach, subsequent layers can learn to combine and weigh the multiscale features as needed. This design enables the network to capture features at multiple scales simultaneously, improving its ability to recognise complex patterns while keeping the computational cost and number of parameters low compared to a classical CNN^19^.

### Scintillator materials, surfaces and detector model

Five nanocomposite materials (LaBr$_3$:Ce-polystyrene, Gd$_2$O$_3$-polyvinyl toluene, LaF$_3$:Ce-polystyrene, LaF$_3$:Ce-oleic acid and YAG:Ce-polystyrene) and four high-density ceramics (GAGG:Ce, GLuGAG:Ce, GYGAG:Ce and LuAG:Pr) are chosen as simulation materials, to enable direct comparison with materials simulated by Wilson et al.^12^. For each material, the physical and optical properties of each scintillator were implemented in GATE using a combination of data obtained from the literature^20–33^. The key parameters include the binding matrix and nanoscintillator chemical composition and nanocomposite loading factor, average nanocomposite density, the refractive index of the matrix and nanoparticles, the average nanoparticle size, scintillation yield and scintillation emission spectrum. The chemical compositions and density are described in GATE’s GateMaterials.db file, while the optical properties are used to calculate the material’s absorption length and Rayleigh scattering length, which are encoded in GATE’s Materials.xml file. The properties of the nanocomposite and ceramic materials are summarised in Tables 2 and 1.

**Table 1:** Properties of several transparent ceramic scintillator materials modelled in this work, adapted from^12^.

**Ceramic**	GYGAG:Ce	GLuGAG:Ce	GAGG:Ce	LuAG:Pr
Peak $\lambda$ (nm)	550	550	530	310
Yield (ph/keV)	50	48.2	70	21.8
R ($\%$ @662 keV)	4.9	7.1	4.9	4.6
1st Decay (ns)	100	84	90	21.4
2nd Decay (ns)	500	148	194	771
$\rho$ (g/cm$^{3}$)	5.8	6.9	6.63	6.73
n (@peak $\lambda$)	1.82	1.92	1.90$^{\star }$	2.03$^{\star }$
$\alpha$ (cm$^{-1}$)	0.10	2.00	3.13$^{*}$	2.86$^{*}$
Refs.	^26–28^	^29,30^	^31,32^	^32,33^

Properties listed with $^{*}$ have been calculated analytically, while those listed with $^{\star }$ were obtained from literature pertaining to the equivalent monocrystalline form of the material. *R* is the energy resolution; $\rho$ is the material density; $\alpha$ is the (optical) linear attenuation coefficient at the peak emission wavelength.

**Table 2:** Physical and optical properties of the nanocomposite scintillator materials modelled in this work, adapted from^12^.

Nanoparticle	LaBr$_{3}$:Ce	Gd$_{2}$O$_{3}$	LaF$_{3}$:Ce	LaF$_{3}$:Ce	YAG:Ce
Matrix	PS	PVT	OA	PS	PS
Load ($\%$ Vol.)	19	4.6	34	50	50
Peak $\lambda$ (nm)	380$^{+}$	550	334	334	550$^{+}$
Yield (ph/keV)	63$^{+}$	22	4.5$^{+}$	4.5$^{+}$	20.3$^{+}$
*R* ($\%$ @662 keV)	2.6$^{+}$	11.4	16$^{+}$	16$^{+}$	11.1$^{+}$
Decay (ns)	16$^{+}$	17	30$^{+}$	30$^{+}$	87.9$^{+}$
$\rho$ (g/cm$^{3}$)	1.81$^{*}$	1.34$^{*}$	2.59$^{*}$	3.47$^{*}$	2.81$^{*}$
*n* (@peak $\lambda$)	1.69$^{*}$	1.56$^{*}$	1.52$^{*}$	1.65$^{*}$	1.72$^{*}$
$\alpha$ (cm$^{-1}$)	2.00$^{*}$	0.09$^{*}$	2.05$^{*}$	0.15$^{*}$	0.95$^{*}$
Refs.	^20,21^	^21,22^	^21,23,24^	^21,23,24^	^21,25^

Properties marked with an $*$ were been estimated from the volume fractions listed, assuming 9 nm diameter nanoparticles. Properties listed with a $^{+}$ are estimates based on the bulk crystalline equivalent of the nanoparticle. *R* is the energy resolution; $\rho$ is the average nanocomposite density; $\alpha$ is the (optical) linear attenuation coefficient at the peak emission wavelength. PS is polystyrene; PVT is polyvinyl toluene; OA is oleic acid.

Each scintillator is modelled as a rectangular prism, with transverse dimensions (*x*, *y*) of 45.6 mm and an optimal thickness (*z*) of $T_{opt}$ for each material taken from Wilson et al., 2020^11^, which provides the optimal sensitivity for a median error in the localisation of the first point of interaction of less than 5 mm (reflecting the intrinsic compromise between gamma sensitivity and internal self-absorption/Rayleigh scattering of the resulting optical photons). $T_{opt}$ for each material is shown in Table 3.

**Table 3:** Optimal thicknesses $T_{opt}$ for each of the materials included in this study (ceramics denoted (c) and nanocomposites denoted (n)).

Material	$T_{opt}$ (mm)
GAGG:Ce (c)	13.98
GLuGAG:Ce (c)	27.54
GYGAG:Ce (c)	42.63
LuAG:Pr (c)	19.07
Gd$_{2}$O$_{3}$/PVT (n)	62.61
LaBr$_{3}$:Ce/PS (n)	53.78
LaF$_{3}$:Ce/OA (n)	32.66
LaF$_{3}$:Ce/PS (n)	42.60
YAG:Ce/PS (n)	46.79

Both proximal and distal surfaces are modelled as a finely polished optical surface coupled to an array of silicon photodetector pixels via a layer of Meltmount ™optical epoxy resin^12,34^. Dual-sided readout (DSR) is a more expensive option compared to single-sided readout (SSR; either front or back), but it enables the use of a thicker scintillator slab since self-attenuation will not prevent the capture of photons from events interacting far from the one photosensitive face. Dual-sided readout also enables a direct comparison with previous results reported by Wilson et al.^12^. Future work will explore the lower-cost alternative of front and back SSR configurations.

Non-photosensitive faces of the scintillator are coated in non-reflective black paint; these properties are described in GATE’s Surfaces.xml file. Again, this is chiefly to enable direct comparison with Wilson et al.^12^; an alternative of a reflective coating will also be considered in future work.

In the simulation, detector quantum efficiency is set to 100% at the emission wavelength; data from this ideal detector is then used for training the neural networks. Subsequent evaluation of localisation and classification performance is performed for realistic detector quantum efficiency by probabilistically eliminating a fraction of the detected photons. Detector QE is thus evaluated over a range from 100% (ideal) to 30%.

### Generation of training data

**Figure 2:**
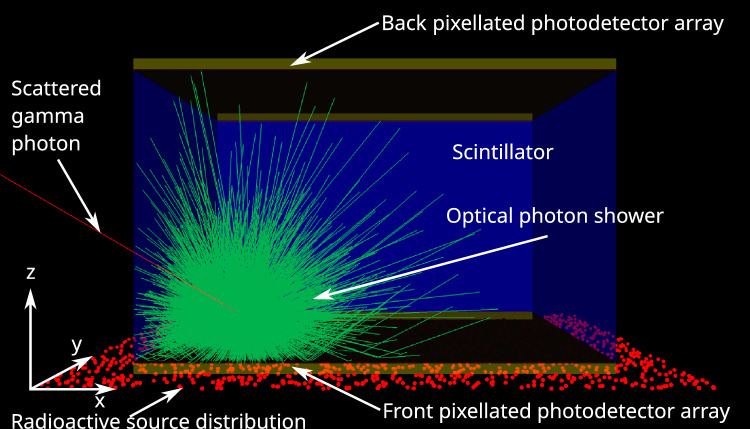
Simulation model showing example scintillation event in GLuGAG:Ce scintillator slab. The layer of red dots on the bottom illustrates the planar radiation source. Gamma photons are shown in red, while optical photons are shown in green. The gamma photon exiting the centre of the scintillation flash to the left represents a Compton-scattered photon escaping from the scintillator, which is one of the most common types of interactions. The front and back faces of the scintillator (bottom and top of the model, respectively) are optically coupled to pixellated photodetector arrays for double-sided readout.

An example simulation (showing a single scintillation event) is shown in Fig. 2.

A planar 60 mm $\times$ 60 mm 511 keV gamma radiation source is placed parallel to the slab, just above the surface of the scintillator/detector slab, with random emission azimuth $az \in [0^\circ , 360^\circ ]$ and elevation $el \in [0^\circ , 90^\circ ]$ (shown as the plane of red dots in Fig. 2). For each material, 50,000 primary particles are simulated. Since further increases beyond this number did not result in any additional improvement in convergence or a reduction in the residual error of the trained networks, this number was selected as it represented the ideal balance between reduced computational overhead and model accuracy.

The GATE simulation records the distribution of optical photons on both the front and back detector planes, quantised into 20$\times$20 pixel arrays. The ground truth location and interaction type for each gamma photon interaction with the crystal are also recorded—most importantly, for the first point of interaction, which is critical in determining the line of response in PET. The amount of energy deposited, the time at which the interaction occurred, and the identity of the parent event are also recorded.

Generation of training datasets for each material took approximately 15-35 core-hours (depending on the material) on an Intel Xeon Gold 6126 CPU.

### Gamma interaction classification

For 511 keV gamma photons, the primary modes of interaction are photoelectric absorption and Compton scattering, with the possibility of multiple Compton scatterings before final absorption or escape (i.e., Compton-photoelectric or Compton-escape)^12^.

The accuracy of the FPoI location estimation is dependent on the interaction mode. Compton scattering followed by a photon escape typically yields a weaker signal compared to photoelectric absorption due to lower energy deposition. Conversely, multi-interaction events, which deposit energy almost simultaneously at multiple locations within the scintillator, generate complex optical photon distributions that degrade the accuracy of FPoI determination. Therefore, classifying the interaction type enables appropriate weighting of events in PET sinogram construction, with higher weight assigned to interactions that provide more reliable FPoI location estimates.

To accomplish this classification, a neural network classifier is designed with three output classes corresponding to one, two or three or more energy depositions. The network architecture comprises of:

- One convolutional layer followed by max pooling (Conv1/MaxPool1)
- One Inception layer plus max pooling layer (Inception1/Maxpool2)
- One flattening layer (FC1)
- Five fully connected layers (FC2-5 plus the output layer)

**Figure 3:**
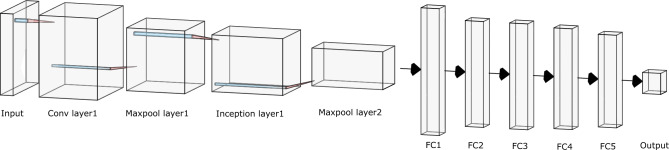
InceptionNet model for event type classification; the network classifies gamma interactions into single, two, or three-plus energy depositions. It consists of an input layer, convolutional and max pooling layers (Conv1/MaxPool1), an Inception layer (Inception1/Maxpool2), a flattening layer (FC1), and five fully connected layers (FC2-5) with an output layer. Batch normalisation and leaky ReLU activation are used throughout.

Batch normalisation is applied after all convolutional layers, including those in the Inception layer. The leaky rectified linear unit (ReLU) serves as the activation function for both convolutional and fully connected layers; Fig. 3 illustrates the network structure, and Tables 4 and 5 detail the network parameters, including kernel size (spatial dimensions of the convolutional filter), stride (pixel shift per operation), padding (zero-padding applied to input borders), and Inception layer specifications, respectively.

**Table 4:** Parameters for the InceptionNet-based network for energy-depositing event classification, including layer sizes, kernel sizes (where applicable), stride, and padding, detailing the network structure from input to output.

Classification network parameter	Layer size	Kernel size	Stride	Padding
Input	$20\times 20\times 2$	–	–	–
Conv1	$20\times 20\times 24$	3	1	1
Maxpool1	$10\times 10\times 24$	2	2	0
Inception1	$10\times 10\times 48$	–	–	–
Maxpool2	$5\times 5\times 48$	2	2	0
FC1	1200	–	–	–
FC2	384	–	–	–
FC3	256	–	–	–
FC4	196	–	–	–
FC5	128	–	–	–
Output	3	–	–	–

**Table 5:** Parameters of the inception layer (inception1) of the classification network.

Inception layer	Branch1	Branch2	Branch3	Branch4
Layer design	$1\times 1$ Conv	$3\times 3$ Conv	$5\times 5$ Conv	Same size maxpool
Input size	$10\times 10\times 24$	$10\times 10\times 24$	$10\times 10\times 24$	$10\times 10\times 24$
Output size	$10\times 10\times 12$	$10\times 10\times 12$	$10\times 10\times 12$	$10\times 10\times 12$

For this task, 50% detector efficiency is assumed.

### FPoI location estimation

Following classification by event type, the $20\times 20\times 2$ optical photon map corresponding to each energy-depositing event is used as an input to the FPoI localisation network. This network is trained to estimate the spatial coordinates of the FPoI within the scintillator. For comparative analysis, two network structures are trained in parallel: a pure CNN and an Inception-based network.

Both network structures share similarities with the classification network, as they all have identical input and output dimensions (three classes in the case of the classification network and three coordinates for spatial location in the case of the regression network). The training parameters, including batch size, learning rate, and optimiser are identical to those used in the classification network.

The CNN is constructed using two convolutional layers, each followed by a max pooling layer, a flattening layer (FC1), and five fully connected layers (FC2-5 plus the output layer). Table 7 lists the network parameters. The InceptionNet variant substitutes an Inception layer for the second convolutional network layer, otherwise maintaining the same structure and parameters as in the classification network. The parameters for this network are listed in Table 6.

**Table 6:** Parameters for the InceptionNet-based network for FPoI localisation, including layer sizes, kernel sizes (where applicable), stride, and padding, detailing the network structure from input to output.

InceptionNet parameter	Layer size	Kernel size	Stride	Padding
Input	$20\times 20\times 2$	–	–	–
Conv1	$20\times 20\times 24$	3	1	1
Maxpool1	$10\times 10\times 24$	2	2	0
Inception1	$10\times 10\times 48$	–	–	–
Maxpool2	$5\times 5\times 48$	2	2	0
FC1	1200	–	–	–
FC2	384	–	–	–
FC3	256	–	–	–
FC4	196	–	–	–
FC5	128	–	–	–
Output	3	–	–	–

**Table 7:** Parameters for the CNN-based network for FPoI localisation, including layer sizes, kernel sizes (where applicable), stride, and padding, detailing the network structure from input to output.

CNN parameter	Layer size	Kernel size	Stride	Padding
Input	$20\times 20\times 2$	–	–	–
Conv1	$20\times 20\times 24$	3	1	1
Maxpool1	$10\times 10\times 24$	2	2	0
Conv2	$8\times 8\times 48$	3	3	0
Maxpool2	$4\times 4\times 48$	2	2	0
FC1	768	–	–	–
FC2	256	–	–	–
FC3	256	–	–	–
FC4	196	–	–	–
FC5	128	–	–	–
Output	3	–	–	–

### Training

The networks are trained using randomly shuffled batches of 2000 samples. A learning rate of 0.001 per epoch is applied; a range of other learning rates was also evaluated, however this parameter did not significantly change the performance of the resulting networks.

A total of 21 datasets were simulated and subsequently divided into training, validation, and testing subsets. Twenty of these datasets were combined and randomly mixed, with 80% used for training and the remaining 20% reserved for validation. The final, completely independent dataset was held out and used exclusively for testing to ensure an unbiased evaluation of model performance. For the localisation network, mean squared error (MSE) is used as the loss function, while the classification network employs cross-entropy loss. Both networks are trained using the Adam optimiser with a weight decay factor of 10$^{-3}$.

The network typically converges within 500 epochs, with a maximum limit set at 800 epochs. During training, network performance is continuously monitored. An early stopping mechanism is implemented, halting the training process when accuracy starts to decrease after reaching a maximum (which typically occurs between 500-600 epochs) to prevent overfitting.

Training of the classifier network takes an average of approximately 19 minutes and the localisation network approximately 25 minutes per dataset on an Intel Xeon E-2488 CPU with an NVidia A2 GPU.

### Model evaluation and metrics

For the classification network, each event’s output is compared to the ground truth and categorised as correct (output matches ground truth) or incorrect.

For the localisation networks (both CNN and InceptionNet), the Euclidean distance between the estimated FPoI and the ground truth is evaluated, as well as the components of this error in depth (i.e. depth of interaction) and lateral displacement directions are evaluated and plotted as a function of detector quantum efficiency. A more detailed statistical description of the error distributions is provided for a detector with quantum efficiency of 50%, including the minimum, maximum, mean, standard deviation, median and interquartile range.

Finally, the dependence of error on the ground truth position within the scintillator slab is evaluated as a heatmap in both the *xy* (lateral) and *yz* (depth) planes.

Inference (i.e. execution of the trained network on the evaluation dataset) takes less than one minute for each evaluation dataset, on the same Intel Xeon CPU / NVidia GPU used for training.

## Results and discussion

### Classifation network

Results for the classification network are shown in Table 8.

**Table 8:** Classification accuracy for events involving one, two and three or more energy depositing interactions with the scintillator (ceramics denoted (c) and nanocomposites denoted (n)); “**M T**” denotes true positives for an *M*-energy-deposition event, i.e. the event was correctly classified, “**N F**” denotes false classification as an *N*-energy-deposition event (where $M \ne N$).

Material	Classification								
	1-dep			2-dep			3+-dep		
	1 T	2 F	3 F	2 T	1 F	3 F	3+T	1 F	2 F
GLuGAG:Ce (c)	90.3	7.2	2.5	80.9	15.7	3.4	67.2	26.2	6.6
GYGAG:Ce (c)	91.1	6.4	2.5	79.9	16.1	4	66.8	25.6	7.6
GAGG:Ce (c)	90.4	6.6	3	77.6	18.1	4.3	67.9	23.3	8.8
LuAG:Pr (c)	90.5	7	2.5	78.8	17.5	3.7	67.7	24.1	8.2
LaBr$_3$:Ce/PS (n)	90.1	7.5	2.4	81.4	16.5	2.1	67.5	24.1	8.4
Gd$_2$O$_3$/PVT (n)	91	6.7	2.3	80.2	15.2	4.6	66.8	23.8	9.4
LaF$_3$:Ce/OA (n)	91.4	7.3	1.3	81.7	16.1	2.2	66.7	25.8	7.5
LaF$_3$:Ce/PS (n)	90.7	6.8	2.5	78.1	17.4	4.5	68.4	24.3	7.3
YAG:Ce/PS (n)	90.3	7.1	2.6	79.1	17.8	3.1	66.4	24.7	8.9

The classifier correctly identified events involving a single energy deposition-either a photoelectric interaction or a Compton interaction followed by the escape of the scattered photon-with an accuracy between 90.1% and 91.4%. Most misclassified single-deposition events were incorrectly labeled as two-deposition interactions (e.g., two Compton interactions followed by photon escape, or one Compton interaction followed by a photoelectric interaction).

The most common interactions involve two energy-deposition steps (Compton-Compton-escape or Compton-photoelectric). For these events, the classifier achieved an accuracy between 77.6% and 81.7%. Misclassifications in this category were most often multi-deposition events mistaken for single-deposition events.

For interactions with three or more energy depositions, the accuracy dropped to 66.4–68.4%, with most errors being misclassified as single-deposition events.

The classification network’s performance was largely independent of the scintillator type (nanocomposite or ceramic) and the specific material used.

### Localisation networks

**Figure 4:**
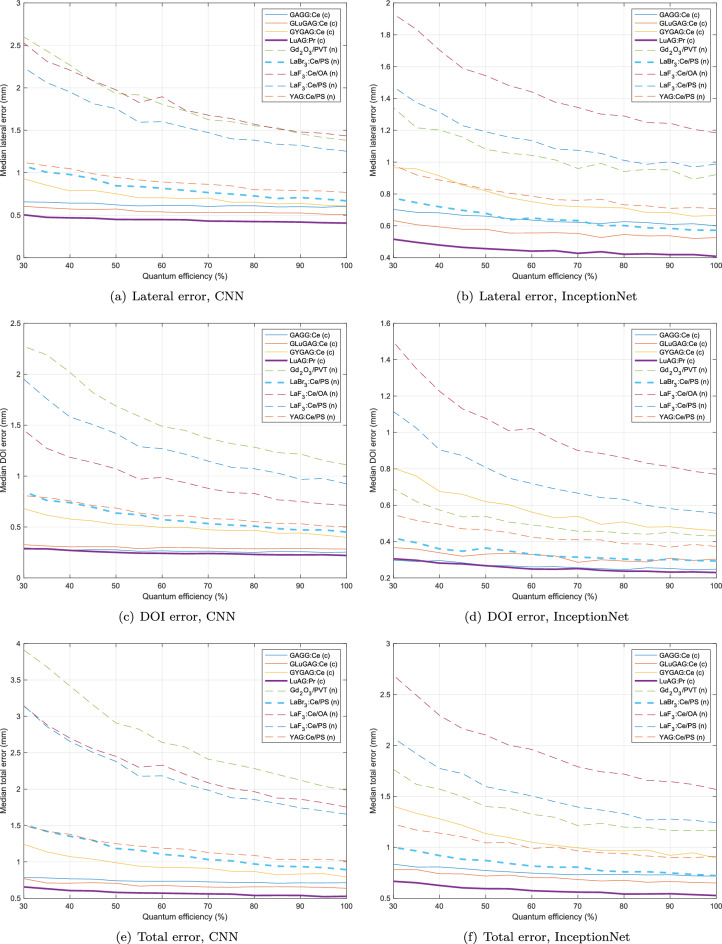
Error in lateral offset, depth (DOI) and total error for each material as a function of detector quantum efficiency, for CNN localisation network (left) and InceptionNet (right). The best performing ceramic and nanocomposite materials are shown in bold (LuAG and LaBr$_3$:Ce/PS, respectively).

Localisation accuracy is evaluated as a function of detector quantum efficiency for each material and localisation network, and the results are plotted in Fig. 4. Localisation accuracy is shown both as overall Euclidean distance and decomposed into error in depth (i.e. depth of interaction) and lateral displacement error (i.e. the radial offset from the projection of the ground truth and estimated position in the *xy* plane).

**Figure 5:**
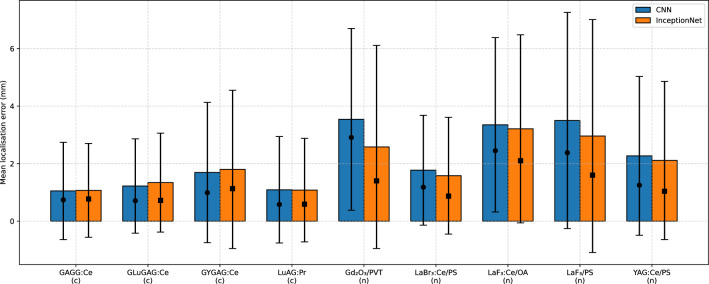
Localisation error comparison across all materials and networks. Confidence intervals are $\pm 2\sigma$ ( 95%).

The first quartile, median, third quartile, mean, and standard deviation of the error (total Euclidean distance) between the ground truth FPoI (obtained directly from GATE) and the estimated FPoI are summarised in Table 9 and Fig. 5 for all nanocomposite and ceramic scintillators.

**Table 9:** Summary of overall localisation accuracy, evaluated across the 3000 events in the test dataset with detector quantum efficiency of 50%. For each scintillator material (ceramics denoted (c) and nanocomposites denoted (n)), localisation network (CNN or InceptionNet), and the first quartile, median, third quartile, mean and standard deviation of the error (Euclidean distance) between the true first point of interaction and the estimated FPoI are listed.

Material	Network	Error (mm)				
		Q1	Median	Q3	$\mu$	$\sigma$
GAGG:Ce (c)	CNN	0.50	0.74	1.11	1.05	1.69
	IncNet	0.51	0.77	1.16	1.07	1.63
GLuGAG:Ce (c)	CNN	0.42	0.71	1.36	1.22	1.64
	IncNet	0.42	0.72	1.60	1.34	1.72
GYGAG:Ce (c)	CNN	0.68	0.99	1.67	1.69	2.44
	IncNet	0.75	1.13	1.80	1.80	2.75
**LuAG:Pr (c)**	**CNN**	**0.34**	**0.58**	**1.12**	**1.09**	**1.85**
	**IncNet**	**0.35**	**0.59**	**1.14**	**1.08**	**1.80**
Gd$_2$O$_3$/PVT (n)	CNN	2.00	2.91	3.94	3.54	3.16
	IncNet	0.70	1.40	3.04	2.58	3.53
**LaBr**$_3$:**Ce/PS (n)**	**CNN**	**0.78**	**1.18**	**1.99**	**1.77**	**1.91**
	**IncNet**	**0.44**	**0.87**	**1.95**	**1.58**	**2.03**
LaF$_3$:Ce/OA (n)	CNN	1.63	2.45	3.92	3.35	3.03
	IncNet	1.32	2.10	3.88	3.21	3.27
LaF$_3$/PS (n)	CNN	1.76	2.38	3.57	3.50	3.76
	IncNet	0.95	1.60	3.32	2.96	4.05
YAG:Ce/PS (n)	CNN	0.84	1.25	2.58	2.27	2.76
	IncNet	0.57	1.04	2.57	2.11	2.75

The best-performing materials are indicated in bold (for both CNN and InceptionNet localisation networks).

For the transparent ceramic materials with 50% detector quantum efficiency, InceptionNet achieved a median error of between 0.59 mm (LuAG:Pr) and 1.13. mm (GYGAG:Ce), with corresponding mean errors of between 1.08 and 1.80 mm. For the CNN, median error was between 0.58 mm (LuAG:Pr) and 0.99 mm (GYGAG:Ce), with corresponding mean errors between 1.09 mm and 1.69 mm. For the nanocomposite scintillator materials, InceptionNet achieved a median error of between 0.87 mm (LaBr$_3$:Ce/PS) and 2.10 mm (LaF$_3$:CE/OA), with corresponding mean errors of between 1.58 mm and 3.21 mm. The CNN achieved errors in the range 1.18 mm (LaBr$_3$:Ce/PS) to 2.91 mm(Gd$_2$O$_3$/PVT) and corresponding mean errors from 1.77 mm to 3.54 mm. Performance of the CNN and InceptionNet was generally very similar for the transparent ceramic materials, while for the nanocomposite materials, InceptionNet offered an average 46% improvement in accuracy over the CNN, suggesting that it is better able to model the effects of Rayleigh scatter and self-attenuation compared to the plain CNN. This observed performance advantage aligns with the theoretical expectation that InceptionNet, through its use of parallel convolutional filters with varying kernel sizes, is better suited for extracting multi-scale features. In this study, the illumination features produced by different scintillators exhibit variations in both spatial extent and intensity. Unlike conventional CNN architectures, which utilise a single kernel size per layer, InceptionNet employs multiple kernel sizes within the same module. This design enables the network to capture a broader and more representative set of features across different spatial scales, resulting in improved accuracy in locating the FPoI within the scintillator-induced illumination pattern.

The best-performing nanocomposite and ceramic materials are LaBr$_3$:Ce/PS and LuAG:Pr, respectively, achieving the best performance amongst the respective scintillator types with both the CNN and InceptionNet.

**Figure 6:**
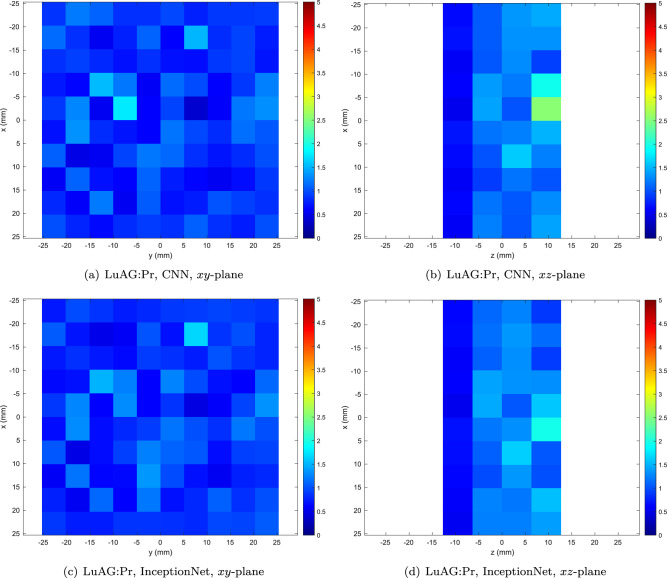
Heatmap of mean total Euclidean error (in mm) as a function of position within the detector for the best-performing ceramic material (LuAG:Pr).

Heatmaps illustrating the spatial distribution of errors in the *xy* (normal to the beam) and *xz* (depthwise) planes for the best-performing transparent ceramic material (LuAG) are shown in Fig. 6. In the *xy* plane, error magnitude is approximately uniform across the detector face, while in the *xz* plane, errors are clearly lowest at the shallowest depth in the scintillator, since these photons are subject to the least self-absorption and scattering.

**Figure 7:**
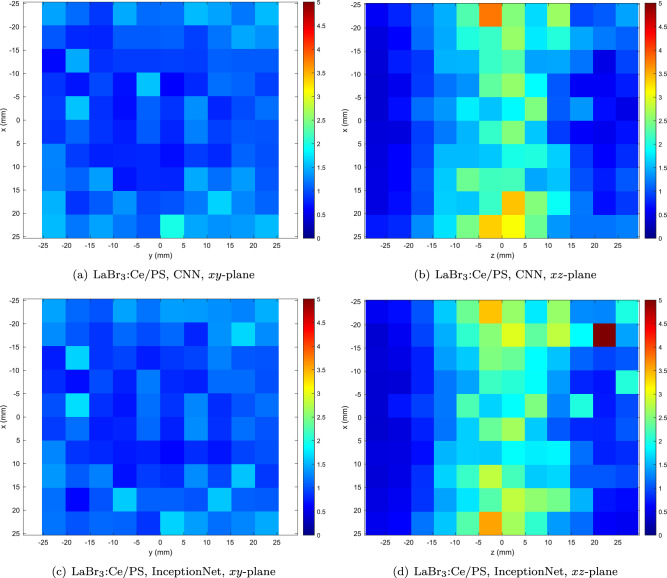
Heatmap of mean total Euclidean error (in mm) as a function of position within the detector for the best-performing nanocomposite material (LaBr$_3$:Ce/PS).

Heatmaps illustrating the spatial distribution of errors in the *xy* and *xz* (i.e. depthwise) planes for the best-performing nanocomposite material (LaBr$_3$:Ce/PS) are shown in Fig. 7. In the *xy* plane, error magnitude is slightly higher around the periphery of the detector face, while in the *xz* plane, errors are clearly lowest near the front and back surface of the scintillator block, and highest near the centre. These effects are more pronounced than in the higher-density LuAG scintillator. The effect seen at the periphery is a result of the non-reflective edge coating absorbing some of the photons emitted by interactions in this region, reducing the amount of available information for inferring the point of interaction. Despite this loss of information, the shape of the photon distribution is preserved; use of a reflective coating is not expected improve performance due to the ambiguity introduced by reflected (especially multiply-reflected) photons^35^.

Higher localisation error in the centre of the scintillator block (in the *z* direction) is a result of the optimal slab thickness being greater (as per^12^) in nanocomposites compared to the ceramic materials. Photons resulting from interactions at this point are subject to the greatest degree of self-absorption and scattering, leading to the relatively high error. Further reductions in this error in nanocomposite materials may be achieved by reducing the size of the nanoparticles and by reducing the difference between the refractive indices of the nanoparticle and the matrix.

Heatmaps for each of the other materials are included in the Supplementary Materials.

### Implications

Accurately estimating the number of energy-depositing events in a monolithic scintillator is of significance for imaging applications, especially PET and hybrid Compton-PET. The quality of information contained in the event is lower with each additional Compton interaction, as it makes the localisation problem more difficult (either for classical approaches or for the neural networks). Correct classification of the event by interaction type would allow the contribution of each component to (for example) a PET sinogram to be weighted according to the number of energy depositions, which should result in a higher quality image. This remains an interesting area for future investigation.

In a typical clinical PET image, 30-40% of the detected annihilation photons have scattered in the patient before reaching the detectors^36^. Of the remaining unscattered photons, the majority that interact with the detector do so via a Compton scattering process (typically more than 90% of the gamma photons interacting with the nanocomposite scintillators and 70–85% for ceramics—see Table 3.3 and 3.4 in^37^). Scattering within the patient can be largely corrected using one of the well-known scatter correction techniques. However, photons which deposit energy within the detector via Compton interaction still carry valuable information. Although they produce a weaker signal compared to a photoelectric interaction (since less energy is deposited), if the first point of interaction can be accurately localised, the line of response is just as valuable as a photoelectric interaction. By classifying detected events according to the number of interactions, we expect that it is possible to extract some useful direction-of-arrival information that would be discarded if we used a simple energy window to filter out only photoelectric events. Gamma photons which have only undergone a single Compton interaction in the detector followed by the escape of the scattered photon will convey the most useful information as these most closely resemble a photoelectric interaction from a line-of-response perspective; however, even multi-interaction energy deposition events can provide useful information (albeit with further degradation in localisation accuracy). Whether or not this is worthwhile is largely dependent on the injected activity and patient/subject dimensions - so especially for small-animal PET, where the scatter fraction is expected to be low and the injected activity is also limited, it would be preferable to utilise these detected events if possible. Our approach provides the option of doing so if desired, providing more information about the nature of each event than can be provided by energy deposition alone.

The accuracy with which the FPoI can be localised using both neural networks significantly improves compared to the classical least-squares error-minimisation approach used by Wilson et al., in which the median and mean errors were 1.2 mm and 2.3 mm for LaBr$_3$:Ce/PS (compared with 0.87 mm and 1.58 mm obtained here), and 0.7 mm and 1.5 mm for LuAG:Pr (compared with 0.58 mm and 1.08 mm).

The improvement relative to the classical approach is most significant for the nanocomposite materials, with the InceptionNet results approaching or exceeding the performance of some of the transparent ceramic materials. This has important economic implications, since the use of nanocomposites, which can often be synthesised at room temperature, has the potential to significantly reduce the cost of detector manufacture relative to those which utilise conventional monocrystalline scintillator materials.

Further cost reductions in the detector can be achieved if readout is limited to single-sided - either front- or back-face. The optimal thickness will need to be recalculated for such a system, but will be thinner (and hence less sensitive) if optimised using the same metric as the dual-sided read out model. While single-sided readout was previously explored by Wilson et al. using classical optimisation-based localisation, this will be the subject of a future investigation as we progress towards prototype implementation.

Translation to practical implementation faces several challenges - most significantly, the problem of achieving high uniformity in a large volume of nanocomposite or transparent ceramic material. Fortunately, neural networks such as the InceptionNet and CNN models used in this research can be fine-tuned via transfer learning using experimental measurements following training with simulation-based data. This process would entail using a collimated source of 511 keV photons, and directing it perpendicular to the *xy*, *yz* and/or *xz* planes. Depth of interaction cannot be controlled, so only two dimensions of the ground truth can be controlled simultaneously; however, by directing photons normal to at least two of these planes, and stepping the beam position through a series of different positions in those planes, all dimensions can be covered.

It is also challenging to achieve high and uniform optical transparency in nanocomposites. The critical parameter in ensuring sufficient transparency for fabrication of thick scintillator blocks is achieving a consistently small nanoparticle size. This is a rapidly evolving field, and new nanocomposites are frequently being reported in the literature (e.g.^38^). We expect that many new and interesting nanocomposite scintillators will emerge in coming years, to which our localisation and classification networks will be well suited.

In addition to the nanocomposite and transparent ceramic materials discussed in this work, there are other promising low-cost materials to which our method would be applicable, including novel Perovskite, glass and glass-ceramic scintillators^39–41^.

## Conclusions

The InceptionNet-based classifier successfully identified over 90.1% of single-energy-deposition events, comprising both photoelectric and Compton-escape interactions, and correctly classified more than 77.6% of all two-deposition events, encompassing Compton–photoelectric and Compton–Compton–escape interactions. Both the InceptionNet and CNN-based localisation networks achieved excellent estimates of the location of the first point of interaction for transparent ceramic materials, while InceptionNet outperformed the CNN by an average of 46% for nanocomposites.

These findings demonstrate that thick nanocomposite scintillators can approach the performance levels of transparent ceramics. The high localisation accuracy achieved, despite greater Rayleigh scattering and self-absorption, confirms the viability of nanocomposites as a scintillator option for PET. Given their significantly lower costs and more benign fabrication and material handling properties compared to both monocrystalline and transparent ceramic materials, these nanocomposites warrant serious consideration for next-generation high-performance PET systems.

## Supplementary Information

Supplementary Information.

## Data Availability

The datasets generated during and/or analysed during the current study are available from the corresponding author (Daniel.Franklin@uts.edu.au) on reasonable request.
