# Supplementary material for: Deep learning based localisation and classification of gamma photon interactions in thick nanocomposite and ceramic monolithic scintillators
Source: Sci Rep. 2025 Aug 5;15:28607. doi: 10.1038/s41598-025-13339-y (PMC12325943; doi:10.1038/s41598-025-13339-y)
Supplement: Supplementary file 1 — Supplementary Information. [file 41598_2025_13339_MOESM1_ESM.pdf]

Deep learning based localisation and classification of gamma photon interactions in thick nanocomposite and ceramic monolithic scintillators: Supplementary Material

Mushen Shen[1], Ragy Abraham[1], Elise Cribbin[1], Harrison Gregor[1], Mitra Safavi-Naeini[2], and Daniel Franklin[1,*]

[1]School of Electrical and Data Engineering, University of Technology Sydney, NSW, Australia
[2]Australian Nuclear Science and Technology Organisation (ANSTO), NSW, Australia
[*]Daniel.Franklin@uts.edu.au

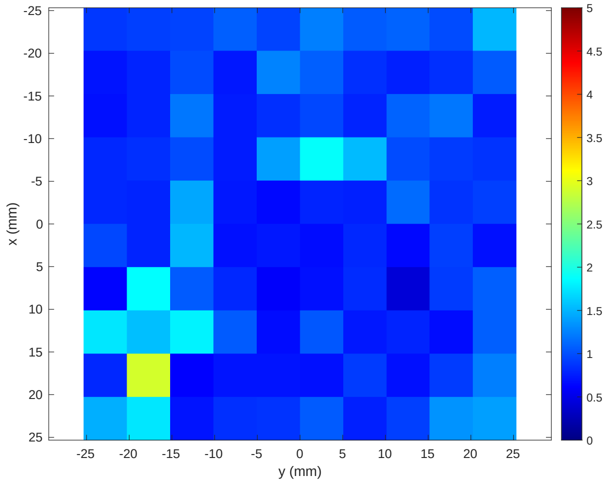

(a) GAGG, CNN, xy-plane

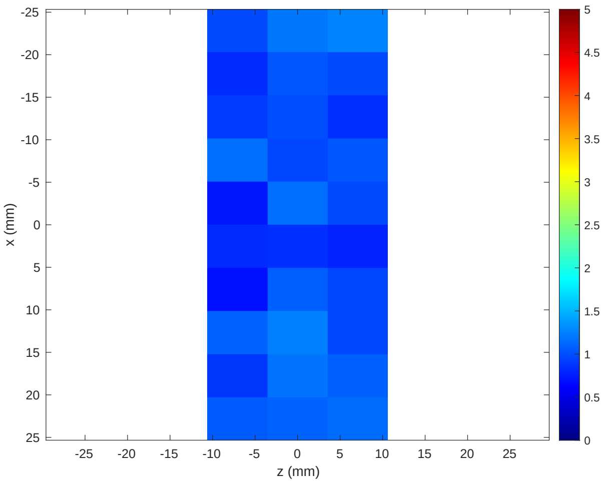

(b) GAGG, CNN, xz-plane

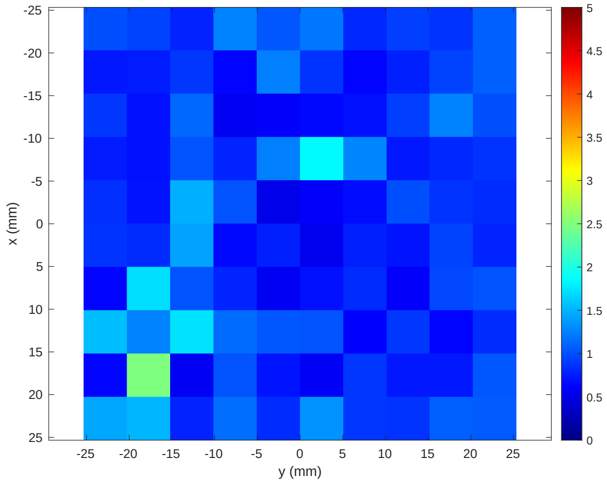

(c) GAGG, InceptionNet, xy-plane

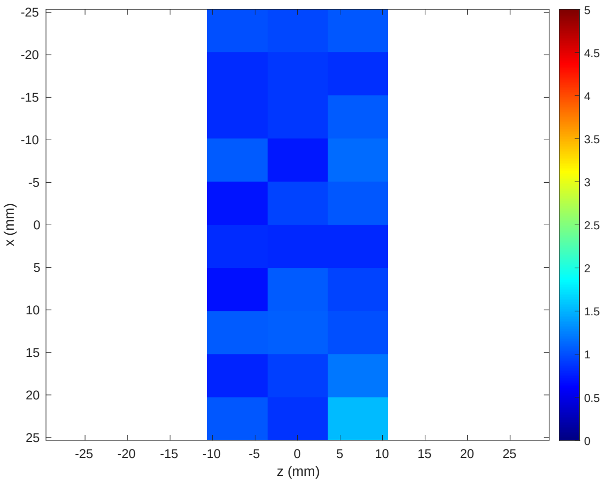

(d) GAGG, InceptionNet, xz-plane

Figure S1. Heatmap of total error as a function of position within the detector for GAGG.

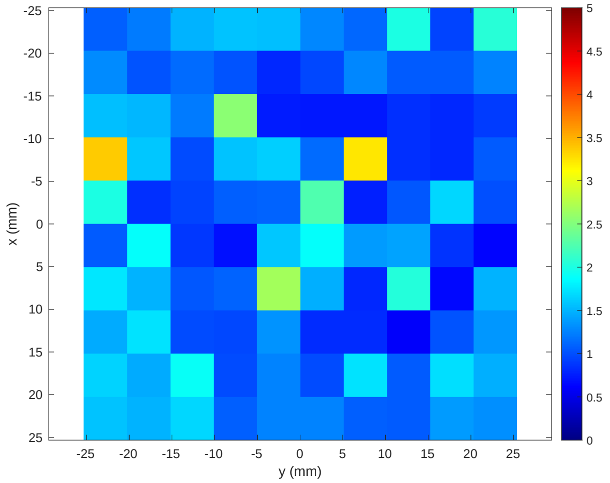

(a) GLuGAG, CNN, xy-plane

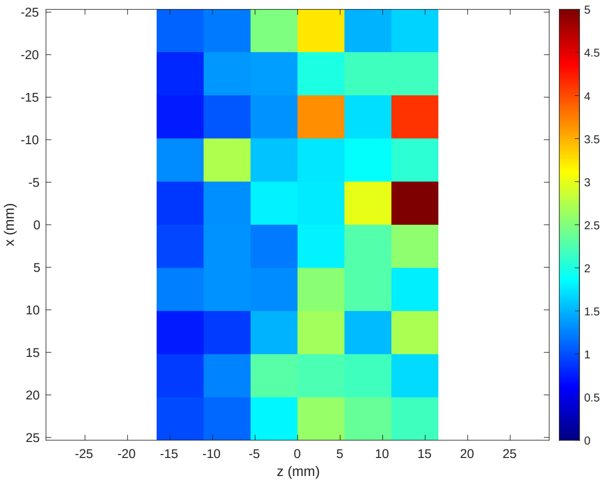

(b) GLuGAG, CNN, xz-plane

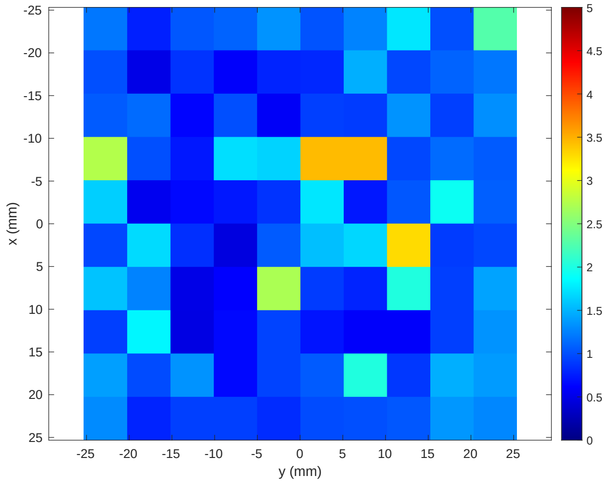

(c) GLuGAG, InceptionNet, xy-plane

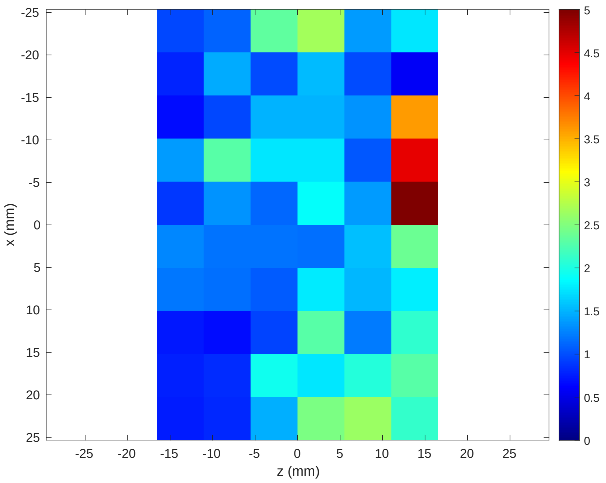

(d) GLuGAG, InceptionNet, xz-plane

Figure S2. Heatmap of total error as a function of position within the detector for GLuGAG.

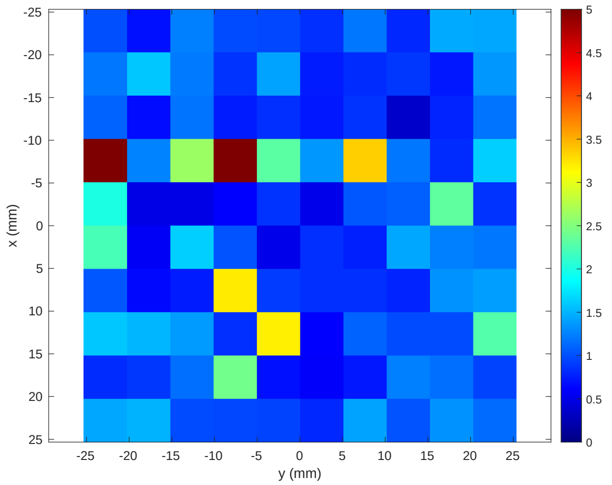

(a) GYGAG, CNN, xy-plane

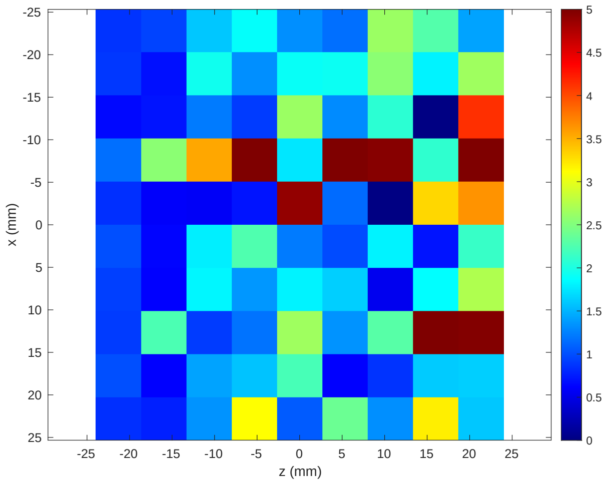

(b) GYGAG, CNN, xz-plane

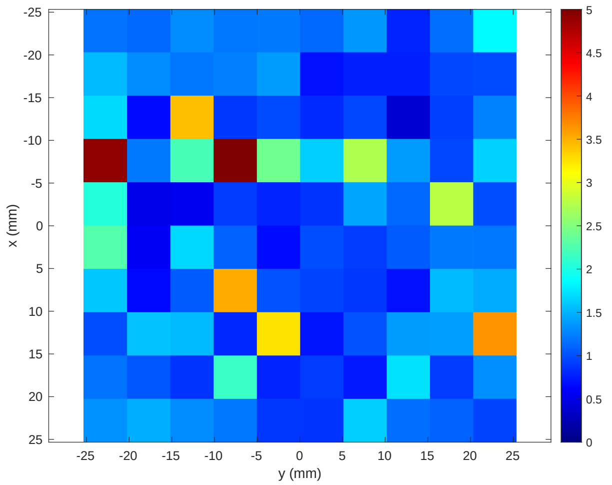

(c) GYGAG, InceptionNet, xy-plane

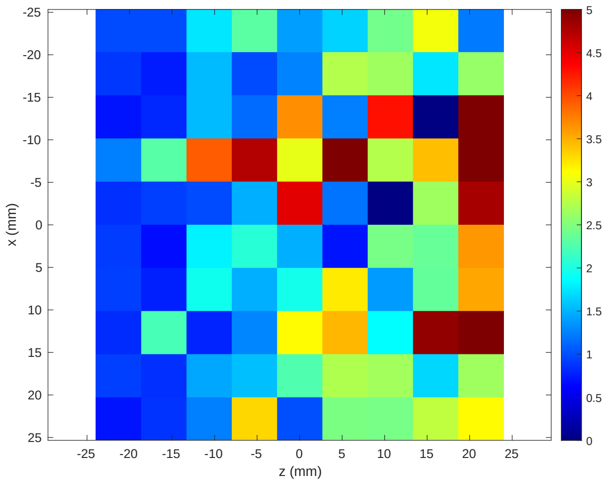

(d) GYGAG, InceptionNet, xz-plane

Figure S3. Heatmap of total error as a function of position within the detector for GYGAG.

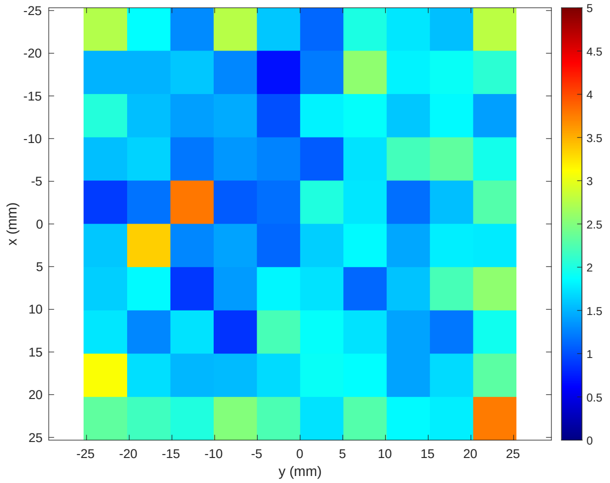

(a) Gd_2O_3/PVT, CNN, xy-plane

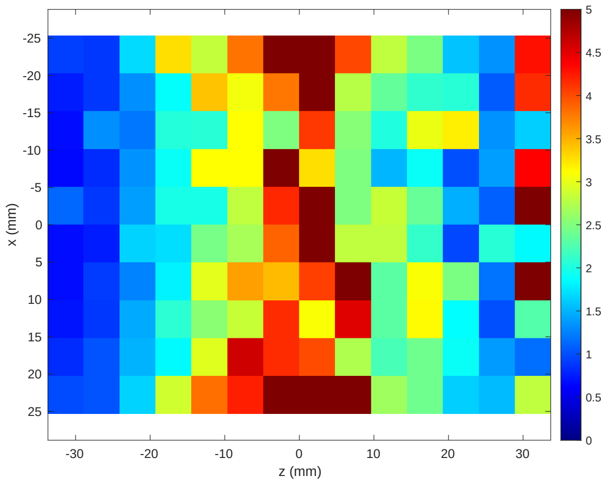

(b) Gd_2O_3/PVT, CNN, xz-plane

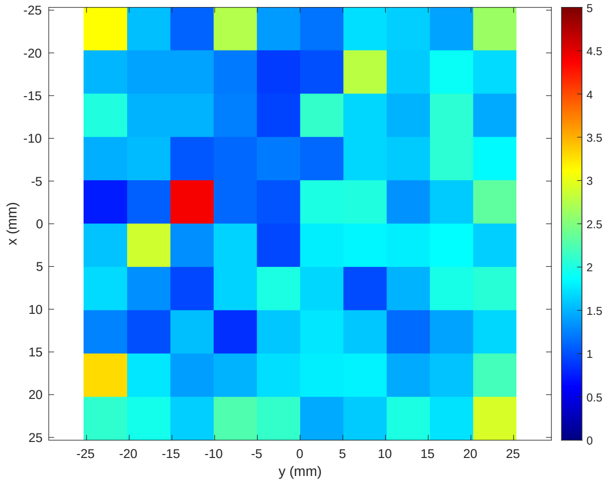

(c) Gd_2O_3/PVT, InceptionNet, xy-plane

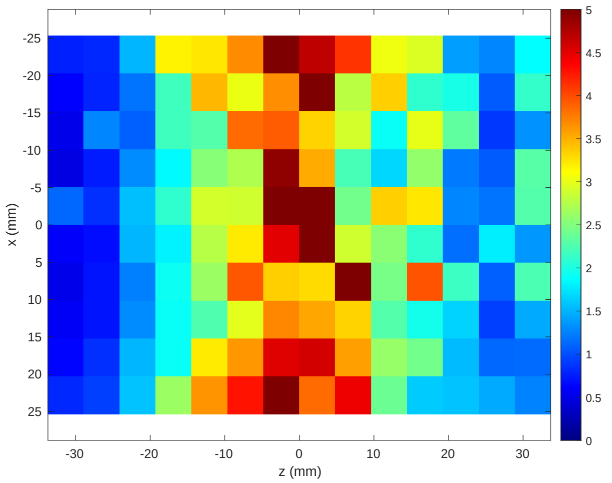

(d) Gd_2O_3/PVT, InceptionNet, xz-plane

Figure S4. Heatmap of total error as a function of position within the detector for Gd_2O_3/PVT.

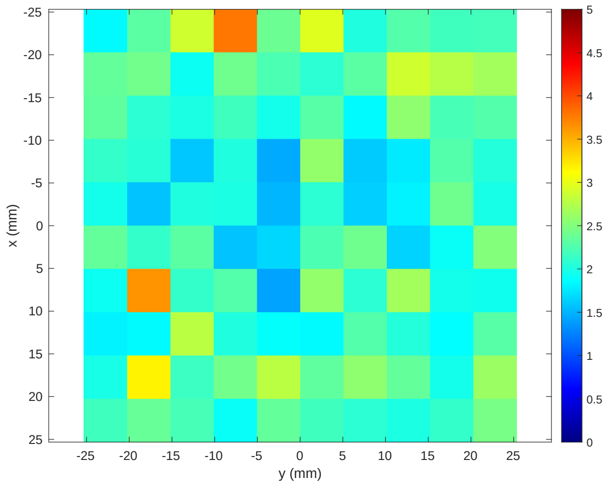

(a) LaF_3:Ce/OA, CNN, xy-plane

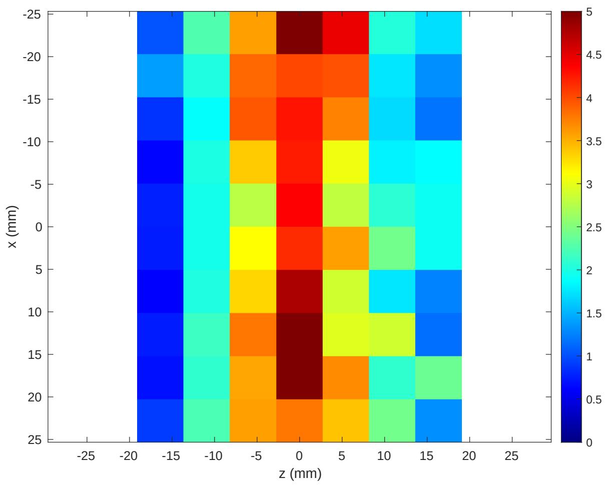

(b) LaF_3:Ce/OA, CNN, xz-plane

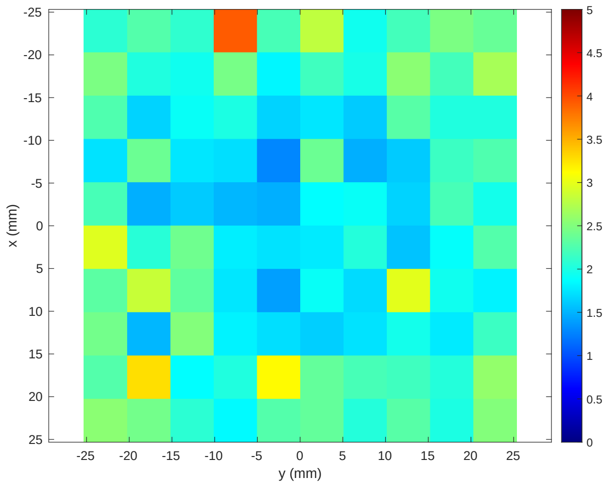

(c) LaF_3:Ce/OA, InceptionNet, xy-plane

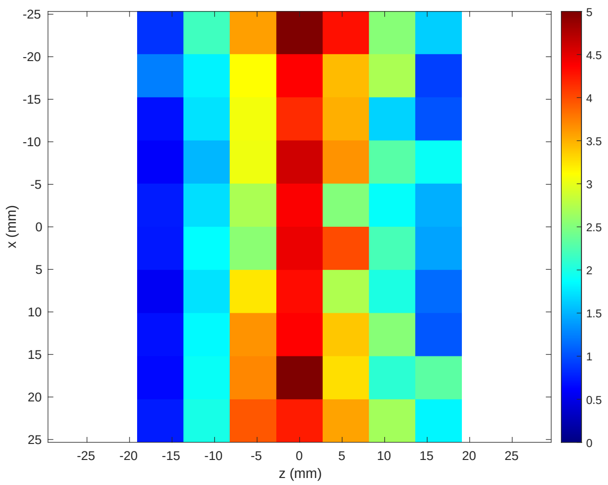

(d) LaF_3:Ce/OA, InceptionNet, xz-plane

Figure S5. Heatmap of total error as a function of position within the detector for LaF_3:Ce/OA.

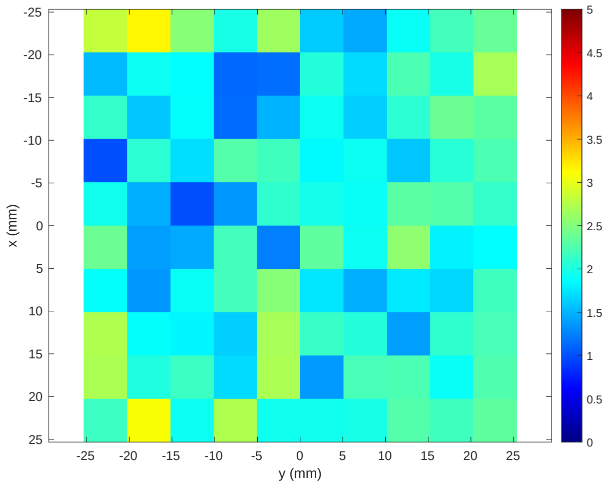

(a) LaF_3:Ce/PS, CNN, xy-plane

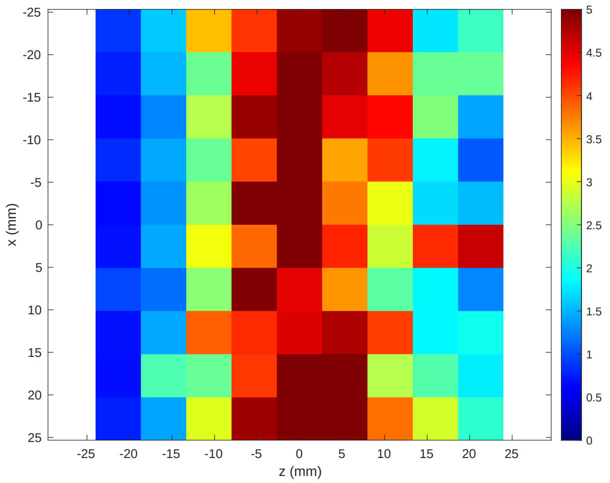

(b) LaF_3:Ce/PS, CNN, xz-plane

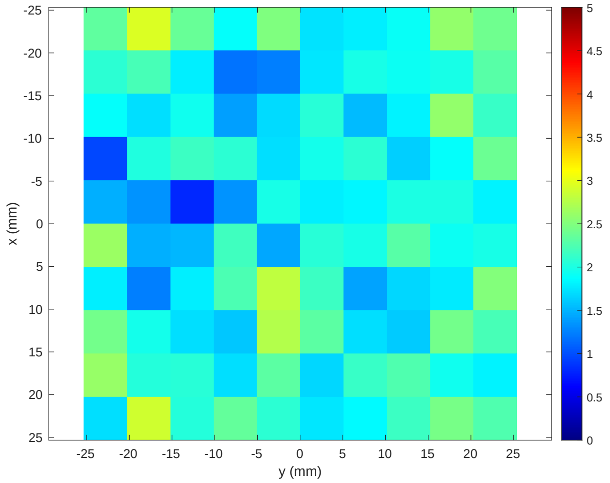

(c) LaF_3:Ce/PS, InceptionNet, xy-plane

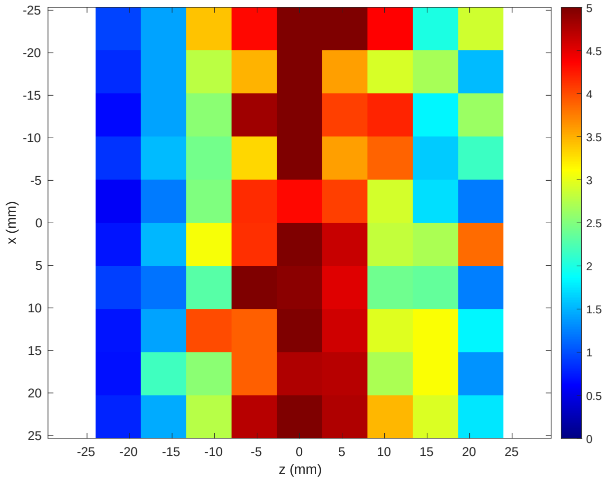

(d) LaF_3:Ce/PS, InceptionNet, xz-plane

Figure S6. Heatmap of total error as a function of position within the detector for LaF_3:Ce/PS.

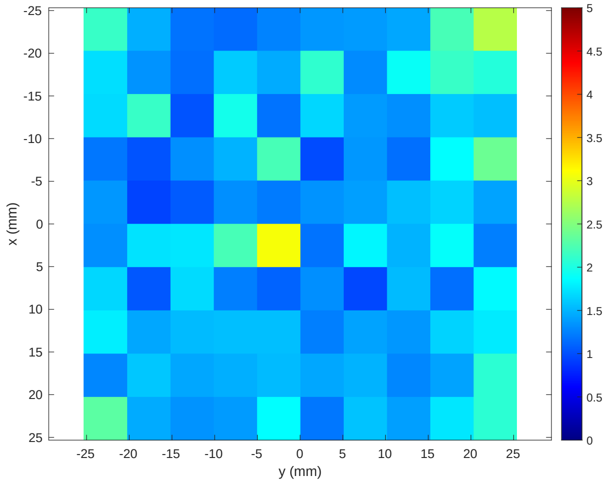

(a) YAG:Ce/PS, CNN, xy-plane

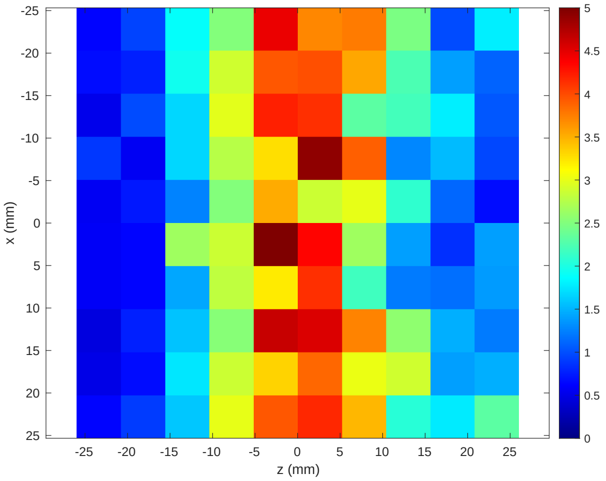

(b) YAG:Ce/PS, CNN, xz-plane

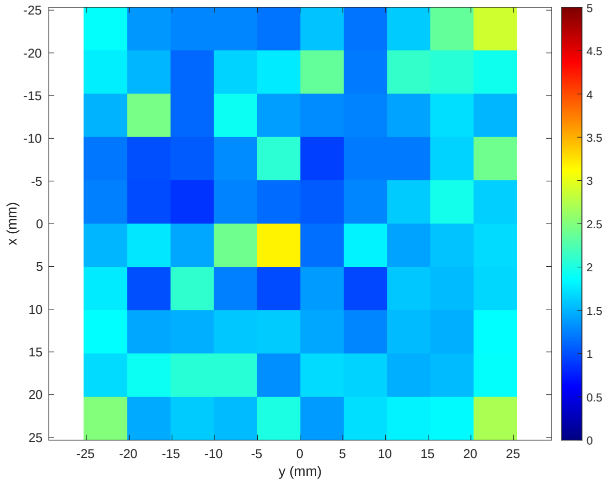

(c) YAG:Ce/PS, InceptionNet, xy-plane

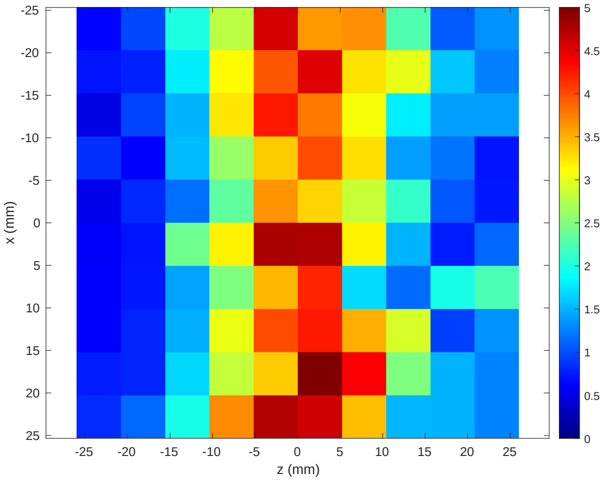

(d) YAG:Ce/PS, InceptionNet, xz-plane

Figure S7. Heatmap of total error as a function of position within the detector for YAG:Ce/PS.
